# Measuring epidermal effects of ostomy skin barriers

**DOI:** 10.1111/srt.12630

**Published:** 2018-11-02

**Authors:** Gary Grove, Tim Houser, Gary Sibbald, Ginger Salvadalena

**Affiliations:** ^1^ cyberDERM inc. Broomall Pennsylvania; ^2^ Toronto Regional (Dermatology) & Wound Healing Clinic Mississauga Ontario; ^3^ Hollister Incorporated Libertyville Illinois

**Keywords:** adhesive devices, erythema, evaporimetry, expert grading, irritation, ostomy barriers, peristomal, skin stripping, skin trauma, TEWL

## Abstract

**Background:**

Ostomy barriers are adhesive devices designed to hold pouching systems to the abdomen and protect the peristomal skin from stoma effluent. The objective of this study was to determine differences in the extent of skin trauma resulting from serially applying and removing two types of ostomy barriers.

**Methods:**

The study was a randomized, prospective, repeated measure trial involving healthy volunteers. The ostomy skin barriers were applied to the abdomen and changed every 3‐4 days over a 17‐day period. Skin observations (erythema, stripping, edge irritation and overall comparisons) were completed by a trained (blinded) observer. Transepidermal water loss (TEWL) measurements were completed by a separate (blinded) technician. TEWL was measured in a designated site and again in the most visually traumatized location at termination.

**Results:**

Statistically significant differences were found between the two test devices in all assessments but visual observation of erythema. Highly significant differences in TEWL were found between the test products when measured at termination from the most visually traumatized sites.

**Conclusions:**

The ostomy barrier with ceramide was significantly less disruptive to the epidermis than the ostomy barrier without ceramide. TEWL measurements were more sensitive to changes in the barrier function of the skin than visual observation of erythema.

## 1 INTRODUCTION

Peristomal skin complications (PSCs) are common occurrences among individuals with a colostomy, ileostomy or urostomy.^1^ The most common cause of PSCs is leakage of stoma effluent onto the skin resulting in peristomal moisture associated dermatitis.^2^ Optimizing ostomy product fit helps to prevent leakage, yet other factors (such as medical adhesive‐related skin injury, sensitivity reactions, and skin infections) can also contribute to irritation in the peristomal area.

Removal of adhesive medical devices is one of the factors known to remove some of the outermost layers of the stratum corneum and lead to elevated transepidermal water loss (TEWL) rates. Computerized evaporimetry is considered the “gold standard” for assessing the extent of stratum corneum barrier disruption.^3^ Since the method was established, there have been numerous reports attesting to the method and relevance of TEWL measurements in evaluating the severity of trauma due to skin stripping.^4^, ^5^, ^6^, ^7^

Measuring TEWL after test strips are removed from the surface of the volar (inside) forearm is a common practice in adhesive tape studies. However, several factors need to be considered when studying the effect of removal of various types of adhesive medical devices to make them relevant to their actual clinical use. Duration of use (dwell time) and the preferred application site are the most critical of these factors.^8^, ^9^ The intended use of ostomy skin barriers is to protect the skin around an abdominal stoma (colostomy, ileostomy or urostomy) from contact with stoma effluent and to adhere the ostomy pouching system to the skin. The primary objective of this study was to determine differences in the extent of normal skin trauma that resulted from serial application and removal of ostomy skin barriers. To maintain clinical relevance, the ostomy skin barriers were applied to the abdomen.

**Figure 1:**
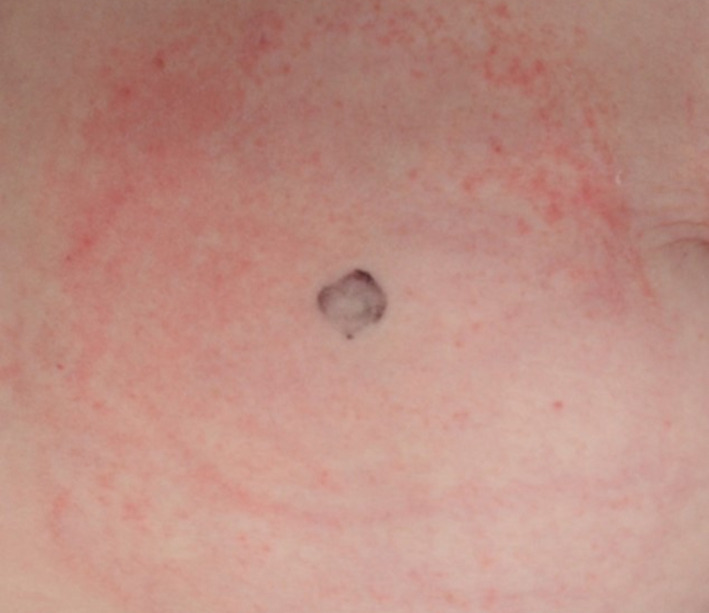
Irregular pattern of irritation

**Figure 2:**
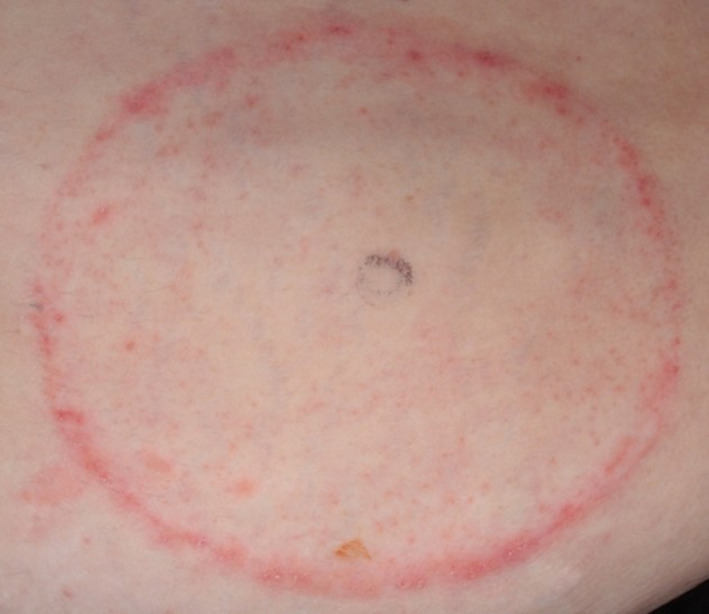
Edge effect

Study methods were slightly modified from those typically used to study the skin effects of adhesive tape removal. Modifications were made to account for use of the ostomy skin barriers on the abdomen, and also for the larger size of ostomy skin barriers in comparison with the typically small test product sizes used in tape removal studies. In prior work, we have observed that variability occurs in the appearance of redness and irritation under ostomy skin barriers which may not be reflected from using a single site of measurement. A typical pattern showing irregularly distributed irritation is shown in Figure 1. There may be a pronounced edge effect along the border of the device with the adjacent non‐covered skin. One particularly clear image of edge effect is shown in Figure 2. Again, this may not be uniformly distributed around the boundary but more often tends to be variable in severity depending upon how the abdomen folds in that region. This may reflect that forces other than adhesive trauma play a role in exacerbating irritation when adhesive ostomy skin barriers are used. To deal with regional variations, we developed an evaluation scheme to divide the test area into 4 zones to be visually scored by the expert grader during each device exchange. Likewise, computerized evaporimetry^10^, ^11^ was used to determine TEWL rates from one designated zone. In those cases where skin trauma was of the severity requiring termination, an additional set of TEWL measurements was taken from the zone that was responsible for the site being terminated.

## 2 MATERIALS AND METHODS

### 2.1 Design

The study was a randomized, prospective, repeated measure trial involving healthy volunteers without a stoma. It was conducted at a single site in the northeastern United States. Institutional Review Board approval was obtained (Allendale Investigational Review Board of RTA Incorporated) and the study was conducted in accordance with ICH‐GCP. Participants were randomized to type of adhesive ostomy barriers, and they came to the study site every 3‐4 days for a total of six visits over the study period of 17 days. Each subject wore two marketed ostomy skin barriers in a paired fashion with a total of five applications and removals during the 17‐day study period. This wear schedule was selected to reflect that commonly followed for ostomy skin barrier change in North America. Ostomy barriers were applied and removed by study staff. Skin observations were completed by a single trained observer while TEWL measurements were completed by a separate technician; each was blinded to the identification of the study barriers and did not see the application or removals of the products. Digital images were taken of participants’ skin following discontinuation or completion of the study.

### 2.2 Test products

Two ostomy skin barriers which are currently marketed in North America (A‐ CeraPlus, Hollister Incorporated, Libertyville, IL; B‐ SenSura Mio, Coloplast Corp, Minneapolis, MN) were selected for use in the study, as shown in Table 1. Both contain an adhesive base along with fluid absorbing hydrocolloids to support adhesion and fluid absorbing capabilities. They may also contain additional functional ingredients which, in the case of Product A, were ceramides. All participants wore both test devices in a paired fashion on contralateral sides of the abdomen that were randomly allocated.

**Table 1:** Test products

Sponsor code	Product name	Product number
A	CeraPlus Skin Barrier	15102
B	Coloplast SenSura Mio	10502

### 2.3 Volunteer candidates

Twenty‐three healthy volunteers with Fitzpatrick scale phototypes I‐III were enrolled into the study. Twenty subjects completed the study; two subjects withdrew consent for further participation and one subject was discontinued from the study after experiencing an adverse event. The 6 male subjects ranged in age from 28 to 60 years with a mean age of 45.3 ± 14.2 years while the 14 female subjects ranged in age 23‐62 years with a mean age of 45.3 ± 11.5 years. Additional details of inclusion and exclusion criteria are listed in Table 2.

**Table 2:** Inclusion and exclusion criteria

Inclusion criteria	Exclusion criteria
Caucasian	Pregnant or nursing mother
Age 18‐65	Menopausal female with hot flashes
Fitzpatrick skin type I, II, or III	Insulin‐dependent diabetes
Sufficient abdominal size to fit two test products	History of mastectomy including lymph node removal
If childbearing potential agrees to use birth control	Clinically significant skin disease (eg, psoriasis, eczema, atopic dermatitis, active cancer)
Agree to refrain from swimming, soaking in hot bath during study	Asthma requiring medication
Agree to refrain from vigorous exercise during study	Known immunological disorders (eg, HIV positive, AIDS, Systemic lupus erythematosus)
Agrees to stop topical products for 3 d prior to study	Cancer treatment within past 6 mo
Agrees to not wash test sites or use topical products other than test products during study	Current use of topical drugs on the abdomen
Does not use anti‐inflammatory medications	Participation in a study involving the abdominal skin in the past 4 wk
	Damaged skin in or around test sites
	Pre‐existing allergy to adhesives or any of the test products

Written consent was obtained from participants prior to their enrollment. Each candidate was instructed to stop the use of all topical products other than their normal cleanser on the abdomen for the 3 days prior to the start of the study. They were also instructed to not wash the test sites or apply any other products to the test sites during the course of this 17‐day trial.

### 2.4 Expert grader assessments

One expert grader made all skin assessments in this study. The assessments were made prior to the first application of the ostomy skin barriers at baseline and approximately 30 minutes post removal of ostomy skin barriers on Days 4, 7, 10, 14 and 17. The skin grader was blinded as to product applications and was not allowed to compare any previous scores. In order to qualify, all assessments at baseline must have been zero. If any of the barriers were partially‐adhered or had fallen off at subsequent study visits, it was recorded and assessments were still made and the barriers were reapplied on schedule.

**Figure 3:**
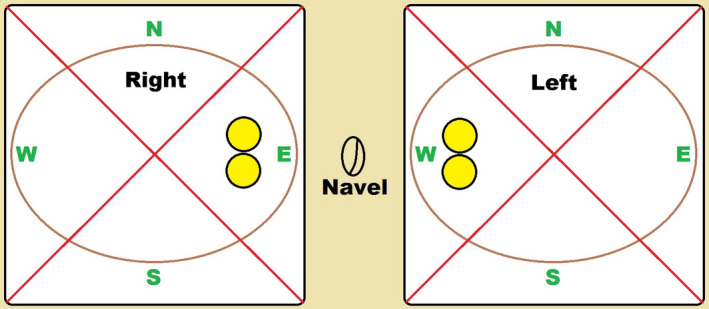
Diagram of measurement sites on the abdomen

All subjects were visually evaluated using the five point ordinal scales for erythema, denudation (skin stripping) and edge irritation where grade 0 was none and grade 4 was severe, with intermediate scores of 1, 2 and 3 representing mild, moderate and marked conditions respectively. Half grades were allowed so that finer distinctions could be made. Zonal grading was performed as per the diagram in Figure 3 **.** Erythema and denudation grading were per‐zone (N, S, E & W) excluding the area at and under the very edge of the device. Edge Irritation grading was per‐zone (N, S, E & W), exclusively for the area at and under the very edge of the device. In addition, the expert grader used a 4 point ordinal scale to provide an overall comparison based on the overall differences in erythema, skin stripping and irritation as a pairwise comparison of two devices. The device considered to be the least traumatic to the skin was given a score of 0 while the opposing test site was given a grade of +1,+2 or +3 if the differences were respectively either slight, moderate or dramatic in comparison.

If any test site zone reached a grade 3 or greater for erythema, 2 or greater for denudation or 4 or greater for edge irritation during the study, treatment on that site was discontinued. Once a site was discontinued, all product application and study assessments were discontinued for both sites with the exception of capturing adverse event information until the irritation was resolved.

### 2.5 TEWL measurements

Transepidermal water loss measurements were obtained with a computerized cyberDERM RG‐1 Evaporimeter^10^, ^11^ equipped with DermaLab TEWL probes (cyberDERM Inc., Broomall, PA, USA). TEWL was measured prior to the first application of the ostomy barriers at baseline (Day 1) and approximately 30 minutes after barrier removal on Days 4, 7, 10, 14 and 17. At each session, duplicate water loss readings were taken from the designated zone within the medial area of each site as indicated by the yellow circles in Figure 3. Additional measurements were made in other zones or edges of a given test site if either the skin was compromised, denuded, eroded or had other signs of irritation that resulted in an early termination.

### 2.6 Statistical analysis

Scores from the four zones within each test site were summed to yield a cumulative score for each of the three parameters (erythema, denudation and edge irritation). If the treatments had been terminated due to severity of irritation, the data from the last observation was then carried forward for that individual in subsequent sessions. In addition to the TEWL measurements obtained from the designated zones of each of the paired sites, any early termination measurements were also taken from the site that showed the most visual signs of irritation regardless of its location.

Data are presented as mean ± SD. *P*‐values < 0.05 were considered statistically significant. Statistical analysis was performed using GraphPad InStat for Windows (GraphPad Software Inc., La Jolla, CA, USA). The Mann‐Whitney test was used for categorical data such as the expert grader's assessments. The Student's paired *t* test was employed for continuous data obtained from TEWL measurements. In those situations where one or both of the sites had been discontinued due to irritation, the last observation was carried forward for both sites for subsequent days.

## 3 RESULTS

**Figure 4:**
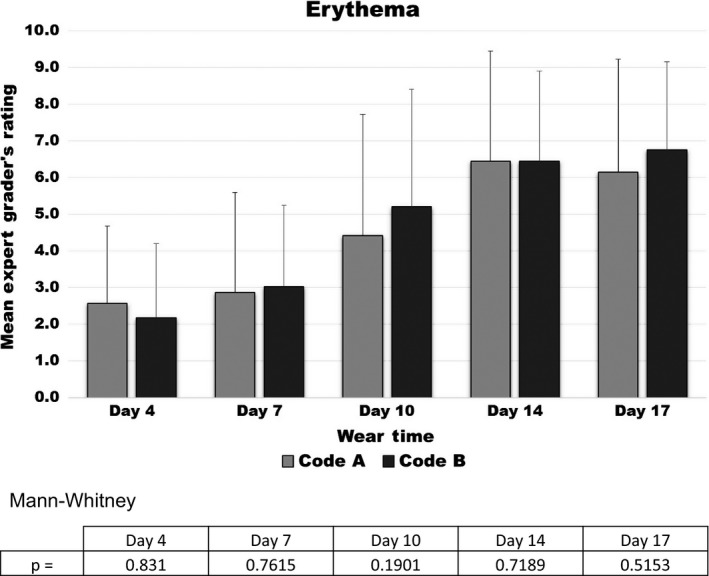
Erythema scores

**Figure 5:**
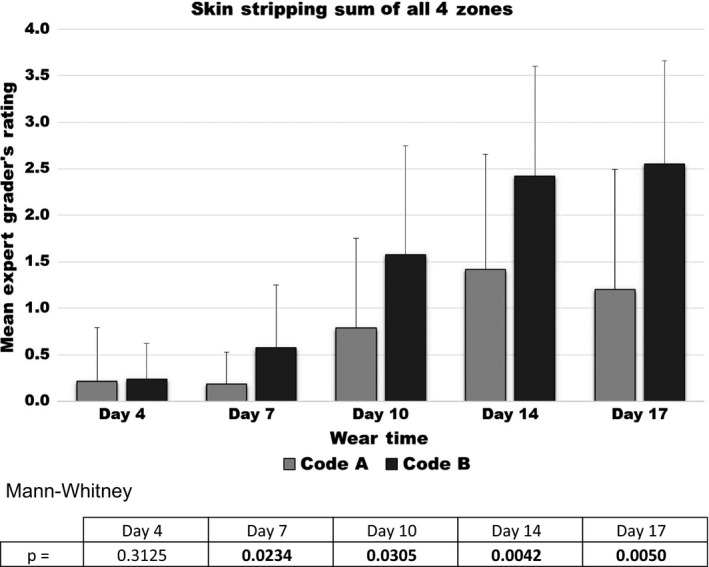
Skin stripping scores

**Figure 6:**
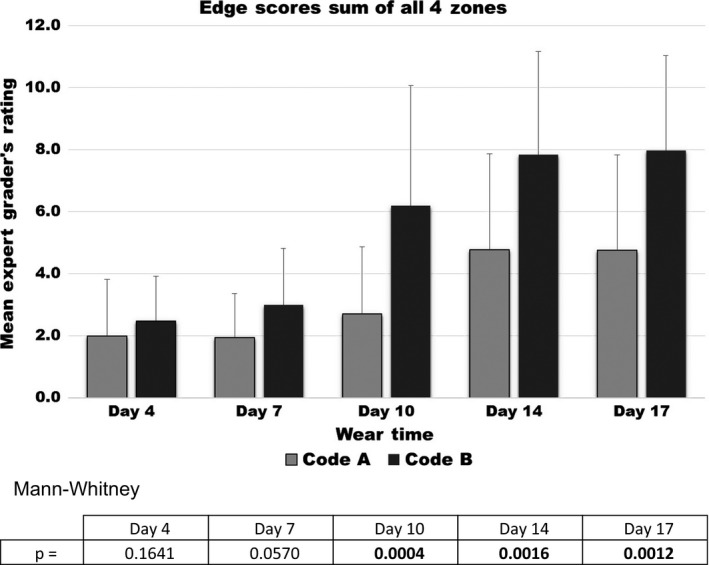
Edge irritation scores

**Figure 7:**
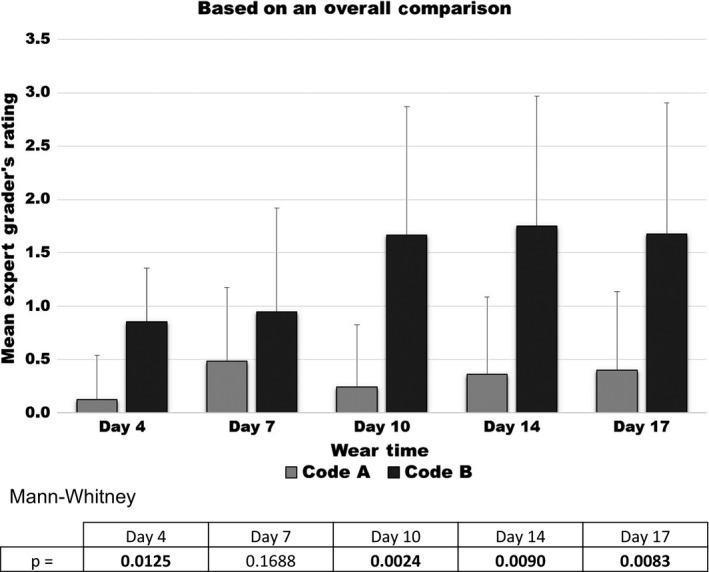
Overall comparison scores

The Expert Grader's Skin scores at the 5 follow‐up observation sessions are displayed in Figures 4, 5, 6, 7 **.** Erythema scores progressively increased over time but in a similar fashion with both test products as shown in Figure 4.On the other hand, significant differences between the test products were observed by the Expert Grader in both the degree of skin stripping (Figure 5) and severity of irritation along the edge (Figure 6). Significant differences between the two test devices were also found based on the overall comparison (Figure 7). In all comparisons, at every time point where significance was achieved, Product A was found to cause significantly less irritation (as evidenced by lower assessment scores) compared to Product B.

**Figure 8:**
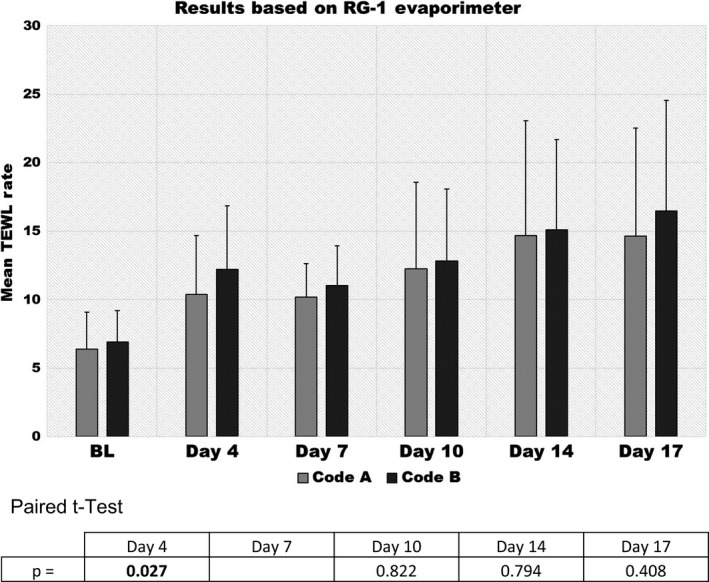
Transepidermal water loss (TEWL) rates at designated site for product A & B

**Figure 9:**
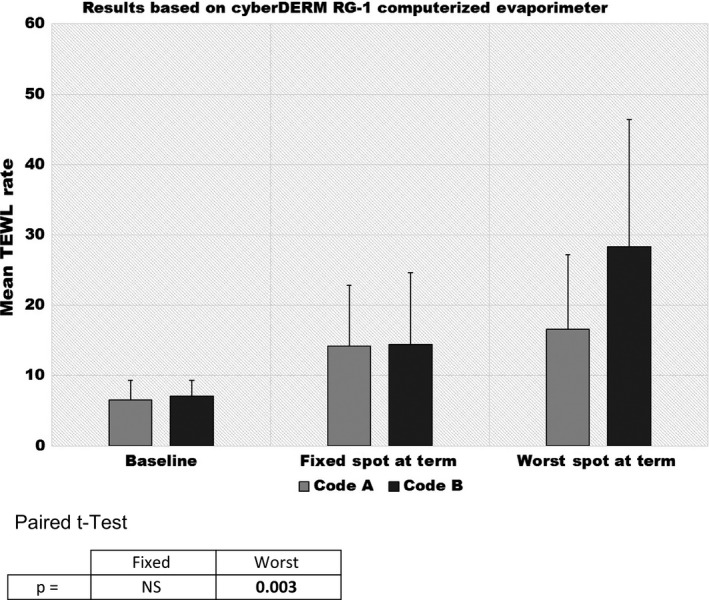
Transepidermal water loss (TEWL) rates at baseline and termination using designated site and worst site

TEWL rates progressively increased over the 17‐day wear period in the designated test sites as shown in Figure 8. Significant differences were found at Days 4 and 7 (*P* = 0.027 and *P* = 0.006) but only directionally thereafter. In striking contrast, highly significant differences were found to exist between the two test products when the analysis was based on TEWL rates measured at the time of termination from the most visually traumatized sites (termed “worst”), as shown in Figure 9. This is most likely due to the practice of discontinuing both sites and carrying forward the TEWL rate obtained from the designated site for those prematurely terminated sites. This practice is referred to as Last Observation Carried Forward (LOCF). This causes the degree of damage that would have resulted from continued use of the problem product to be underestimated later on.^12^, ^13^ The situation is further exacerbated by the fact that in many cases the changes observed by the Expert Grader that were responsible for the site being terminated were not found within the designated site. Thus, it is not surprising that no significant differences were found to exist in the LOCF TEWL readings obtained from the designated sites.

## 4 DISCUSSION

Skin trauma following repeated removal of adhesive medical devices is often measured using visual observation; however, other methods such as TEWL may be more sensitive for the measurement of skin trauma effects. This clinical study was designed to reflect actual use conditions with regard to wear time and application site with healthy individuals. Modifications were made in measurement and analysis to account for size of the product and the potential for irregular patterns of irritation.

Product A was less likely to cause irritation that would lead to an early termination compared to product B. As expected, TEWL rates increased with each of the five device changes that occurred during the 17‐day trial. The TEWL findings are in good general agreement with the Expert Grader's ratings, particularly stripping/denudation. In the case of skin stripping/denudation, the Expert Grader's rating increased progressively over time but much more so in those sites assigned to product B than product A. In the case of Edge scores, the differences are only directional at Day 7 but become significant at all later time points and indicate that this trauma is much more pronounced as time goes on. The overall comparison scores also indicated that there was less irritation with product A than product B and that these differences can be appreciated fairly early on.

Our findings are generally consistent with our understanding that removal of an adhesive device is a form of mechanical insult to the stratum corneum and can result in increased TEWL. The findings of this study suggest the product with ceramide provided some level of protection against the potentially damaging effects of ostomy skin barrier removal in relation to the comparator product. This may be due to the fact that ceramides have dual action: they can bind water and can also act as a lubricant, which may help prevent injury over time.

Some limitations inherent to product study with healthy volunteers are noted. Study participants were naïve to ostomy skin barriers and did not have stomas; thus, their response to the products may not represent what would be expected in actual use of the products. The design did not include a sham control; thus, the cause of the difference between groups may be factors other than the presence or absence of ceramides in Product A. The practice of using LOCF also allows for underestimation of the effects of the worse product.

## 5 CONCLUSIONS

Product A (CeraPlus) was significantly less disruptive to the underlying epidermis when compared to Product B as demonstrated by skin stripping, edge irritation, TEWL and overall comparison grading. This suggests that of the ways to improve ostomy skin barrier function, a particularly good one may be the addition of ceramide.

We feel that applying the ostomy skin devices in a paired wise fashion at the body location where they are intended to be used and monitoring the changes in skin conditions in a standard fashion after each change as was done in this study provides a more accurate measure of skin trauma than previous methods. These modified methods, which take into consideration the heterogeneity of the injury, may be helpful to other researchers conducting skin trauma studies on adhesive devices.
